# Impact of age on the association between cardiac high-energy phosphate metabolism and cardiac power in women

**DOI:** 10.1136/heartjnl-2017-311275

**Published:** 2017-06-12

**Authors:** Maria Nathania, Kieren G Hollingsworth, Matthew Bates, Christopher Eggett, Michael I Trenell, Lazar Velicki, Petar M Seferovic, Guy A MacGowan, Doug M Turnbull, Djordje G Jakovljevic

**Affiliations:** 1 Institute of Cellular Medicine, Newcastle Magnetic Resonance Centre, Medical School, Newcastle University, Newcastle upon Tyne, UK; 2 Cardiothoracic Department, James Cook University Hospital, Middleborough, UK; 3 Faculty of Medicine, University of Novi Sad Novi Sad, Serbia and Institute of Cardiovascular Diseases Vojvodina (Clinic for Cardiovascular Surgery), Sremska Kamenica, Serbia; 4 Department of Cardiology, Clinical Centre of Serbia, Medical School, University of Belgrade, Belgrade, Serbia; 5 Department of Cardiology, Freeman Hospital and Institute of Genetic Medicine, Newcastle University, Newcastle upon Tyne, UK; 6 Research Councils UK Centre for Ageing and Vitality, Newcastle University, UK; 7 Wellcome Trust Centre for Mitochondrial Research, Institute of Neurosciences, Newcastle University, Newcastle upon Tyne, UK; 8 Clinical Research Facility, Royal Victoria Infirmary, Newcastle upon Tyne, UK

**Keywords:** heart, age, metabolism, function

## Abstract

**Objective:**

Diminished cardiac high-energy phosphate metabolism (phosphocreatine-to-ATP (PCr:ATP) ratio) and cardiac power with age may play an important roles in development of cardiac dysfunction and heart failure. The study defines the impact of age on PCr:ATP ratio and cardiac power and their relationship.

**Methods:**

Thirty-five healthy women (young≤50 years, n=20; and old≥60 years, n=15) underwent cardiac MRI with ^31^P spectroscopy to assess PCr:ATP ratio and performed maximal graded cardiopulmonary exercise testing with simultaneous gas-exchange and central haemodynamic measurements. Peak cardiac power output, as the best measure of pumping capability and performance of the heart, was calculated as the product of peak exercise cardiac output and mean arterial blood pressure.

**Results:**

PCr:ATP ratio was significantly lower in old compared with young age group (1.92±0.48 vs 2.29±0.55, p=0.03), as were peak cardiac power output (3.35±0.73 vs 4.14±0.81W, p=0.01), diastolic function (ie, early-to-late diastolic filling ratio, 1.33±0.54 vs 3.07±1.84, p<0.01) and peak exercise oxygen consumption (1382.9±255.0 vs 1940.3±434.4 mL/min, p<0.01). Further analysis revealed that PCr:ATP ratio shows a significant positive relationship with early-to-late diastolic filling ratio (r=0.46, p=0.02), peak cardiac power output (r=0.44, p=0.02) and peak oxygen consumption (r=0.51, p=0.01).

**Conclusions:**

High-energy phosphate metabolism and peak power of the heart decline with age. Significant positive relationship between PCr:ATP ratio, early-to-late diastolic filling ratio and peak cardiac power output suggests that cardiac high-energy phosphate metabolism may be an important determinant of cardiac function and performance.

## Introduction

Heart failure is a clinical syndrome associated with cardiac dysfunction at rest and/or in response to stress. Neubauer proposed that the heart failure is ‘an engine out of fuel’ and diminished cardiac high-energy phosphate metabolism plays an important role in the mechanisms of heart failure.^1^ The heart has one of the largest metabolic demands in the body and it uses chemical energy stored in phosphoryl bonds of metabolites from its primary substrate (ie, fatty acid, carbohydrate and ketone bodies) to maintain its contractile function.^2^ ATP followed by phosphocreatine (PCr) are the two most important high-energy phosphates in the heart.^2^ Derangements in cardiac metabolism deprive the heart of high-energy phosphates required to maintain its function and performance and may cause mechanical failure of the heart.^3^ Studies have also shown that all components of cardiac metabolism (ie, substrate utilisation, mitochondrial oxidative phospshorylation, high-energy phosphate metabolism and PCr:ATP ratio) are impaired in heart failure.^1 4 5^ Experimental studies using various animal models demonstrated the link between cardiac energetics and cardiac function.^6 7^ Glucose 6-phosphate dehydrogenase (G6PD) is a major key enzyme in NAPDH generation, and G6PD-deficient mice exhibited diminished contractile function and high oxidative stress.^6^ Improvement in cardiac metabolism may lead to better cardiac function, whereas diminished cardiac energy metabolism is a cause of heart failure.^6^

Currently, limited number of clinical studies have investigated the association between cardiac high-energy phosphate metabolism and cardiac function and performance. In particular, no study reported the relationship between PCr:ATP ratio and peak cardiac power output, which is proposed to be the best indicator of overall function and pumping capability of the heart, and the strongest predictor of prognosis in heart failure.^8^ Better understanding of the interaction between metabolism and function of the heart may lead to development of new treatment strategies to improve clinical outcomes. Therefore, the aim of the present study is to first, define the effect of age on cardiac high-energy phosphate metabolism and peak cardiac power output and second, assess the relationship between PCr:ATP ratio and cardiac function and performance. Because of a significant gender difference in age-associated changes in cardiac morphology and function,^9^ we designed this study to address the relationship between cardiac metabolism and performance in women.

## Methods

### Study design

This was a single-centre, cross-sectional, observational study.

### Participants

Thirty-five healthy women were recruited as volunteers for the study and grouped according to age (young ≤50 years old, n=20; and old ≥60 years old, n=15). Subjects were included in the study only if they (1) had no history of cardiovascular disease, pulmonary diseases and other chronic diseases and (2) had normal glucose tolerance and lipid profile, normal resting blood pressure, normal ECG and body mass index ≤30 kg/m^2^. Subjects were excluded from the study if they (1) were current or past smokers; (2) were taking any medication known to affect cardiovascular function or (3) were not able to perform maximal graded cardiopulmonary exercise stress test. All participants signed an informed consent according to the Declaration of Helsinki, and the study was approved by the National Health Service North East England—Tyne and Wear South.

### Procedures

All study participants underwent cardiac MRI examination using a 3.0T Philips Intera and a 6-channel cardiac coil (Philips; Best, Netherlands (NL)) with ECG gating. Patients were asked to lie in a supine position and hold their breath while images were obtained in a short axis view. A ViewForum workstation (Philips) was used for cardiac analysis. Epicardial and endocardial borders were manually traced at end-systole and end-diastole on the short axis slices to calculate measures of cardiac function and structure. Contour selection and calculation of left-ventricular mass, systolic and diastolic parameters were performed following previously used protocol.^10 11^

Cardiac high-energy phosphate metabolism was assessed using ^31^P magnetic resonance spectroscopy. Data were collected using a 10 cm diameter ^31^P surface coil (Pulseteq, UK) for transmission/reception of signal. Subjects were placed in a prone position. Shimming was performed using a cardiac triggered, breath-held field map. A slice-selective, cardiac-gated 1-dimensional chemical shift imaging (1D-CSI) sequence was used with a 7 cm slice selective pulse applied foot head to eliminate contamination from the liver, with spatial presaturation of lateral skeletal muscle. Sixteen coronal phase-encoding steps were used, yielding spectra from 10 mm slices (repitition time (TR) = heart rate, 192 averages at the centre of k-space with acquisition weighting and ~20 min acquisition time). Spectral locations were overlaid onto an anatomical image, and the first spectrum arising entirely beyond the chest wall was selected. Quantification of PCr, the γ resonance of ATP and 2,3-diphosphoglycerate (DPG) was performed using the AMARES time-domain fit routine in the jMRUI-processing software. Corrections for saturation, flip angle and blood ATP content were made as published previously.^12^

In addition to cardiac MRI, all participants performed maximal graded cardiopulmonary exercise test on an electromagnetically controlled semirecumbent cycle ergometer. Throughout the exercise test, gas exchange measurement system (Metalyzer 3B, Cortex, Leipzig, Germany) and the bioreactance method (NICOM, Cheetah Medical, Delaware) were used to non-invasively measure gas exchange and central haemodynamics. The bioreactance method accurately measures frequency of relative phase shift of oscillating electronic current across the thorax, and we have recently reported its validity and reliability to assess cardiac output at rest and during exercise.^13–16^ It has greater signal-to-noise ratio by 100-fold compared with the older bioimpedance method and is less susceptible to disruption from excessive movements, adipose tissue and electrode placement.^15^ Throughout the test, 12-lead electrocardiogram (Custo, CustoMed, GmbH, Ottobrunn, Germany) and blood pressure (Tango, SunTech Medical; Morrisville, North Carolina, USA) were recorded. During the exercise test, participants were asked to cycle at 20 W for 3 min as a warm-up period, followed by increase in workload of 10 W per minute, until volitional exhaustion. Cardiopulmonary exercise test was terminated when (1) subject has reached volitional exhaustion, that is inability to pedal at cadence of 50 revolutions per minute; (2) maximal oxygen consumption was achieved, that is there was no further increase in oxygen utilisation despite increase in exercise intensity (watts); (3) respiratory exchange ratio >1.15 or (4) subject voluntarily terminated the test. Cardiac power output, expressed in watts, was calculated as the product of peak exercise cardiac output and mean arterial blood pressure.^8^ Cardiac reserve was defined as the difference between peak and resting cardiac power output. Peak oxygen consumption, as a measure of metabolic response, was defined as the average oxygen uptake during the last minute of exercise.

Physical activity level and number of steps were assessed objectively using a validated portable multisensor array (Sensewear, Bodymedia, Pennsylvania). The monitor was worn for 7 days and was only removed for bathing.

### Statistical analysis and sample size

All statistical analysis was carried out using SPSS V.21.0. Prior to statistical analysis, data were tested for univariate and multivariate outliers using standard Z-distribution cut-offs and Mahalanobis distance tests. Kolmogorov-Smirnov test was used to assess normality of distribution. Differences between age groups were assessed using t-test. Pearson coefficient of correlation was used to assess the relationship between variables. Statistical significance was indicated if p<0.05. Data are presented as mean ± SD unless stated otherwise. A previous study, in which a similar outcome was used, reported a mean difference of 0.24 in PCr:ATP ratio to be significant between younger and older age groups.^17^ To show this difference with at least 70% power at the 5% significance, we needed data for a total of 28 participants (ie, ≥14 participants per age group). We have therefore recruited 35 participants (15 younger and 20 older) for the study.

## Results

Data were normally distributed and not outliers were identified in variables of interests. Participants’ demographic details are presented in table 1. Anthropometric measures were not significantly different between young and old groups except height. Other measures, such as weight, body mass index, body surface area, fat body mass and lean body mass, were not significantly different between the two groups (p>0.05). The level of physical activity (number of steps per day) was also not significantly different between younger and older women (table 1).

**Table 1:** Participant demographic and clinical measures

	All participants	Young (≤50 years old, n=20)	Older (≥60 years old, n=15)	p Value
Age (years)	55.7±14.0 (40–81)	44.4±3.2 (40–50)	70.9±5.7 (63–81)	0.00
Weight (kg)	67.7±11.2 (45–93)	69.2±12.3 (53–93)	65.6±9.6 (45–82)	0.35
Height (cm)	163.0±5.8 (149–175)	166.0±4.8 (155–175)	159.2±4.7 (149–165)	0.00
Body mass index (kg/m^2^)	25.5±3.7 (17.9–30.4)	25.2±4.0 (19–30.4)	25.9±3.4 (18–29.8)	0.59
Body surface area (m^2^)	1.7±0.1 (1.5–2.1)	1.8±0.1 (1.6–2.2)	1.7±0.1 (1.5–1.9)	0.06
Fat body mass (kg)	23.6±9.1 (5.3–34.1)	22.7±10.1 (5.8–34.1)	24.8±7.7 (5.3–31.2)	0.51
Physical activity (steps/day)	11532±5422 (7694–16348)	11578±4985 (7882–16348)	11651±5012 (7694–15854)	0.72

Mean ± SD (range).

p Indicates difference between young and older group.

### The effect of age on cardiac structure, function and metabolism

**Figure 1:**
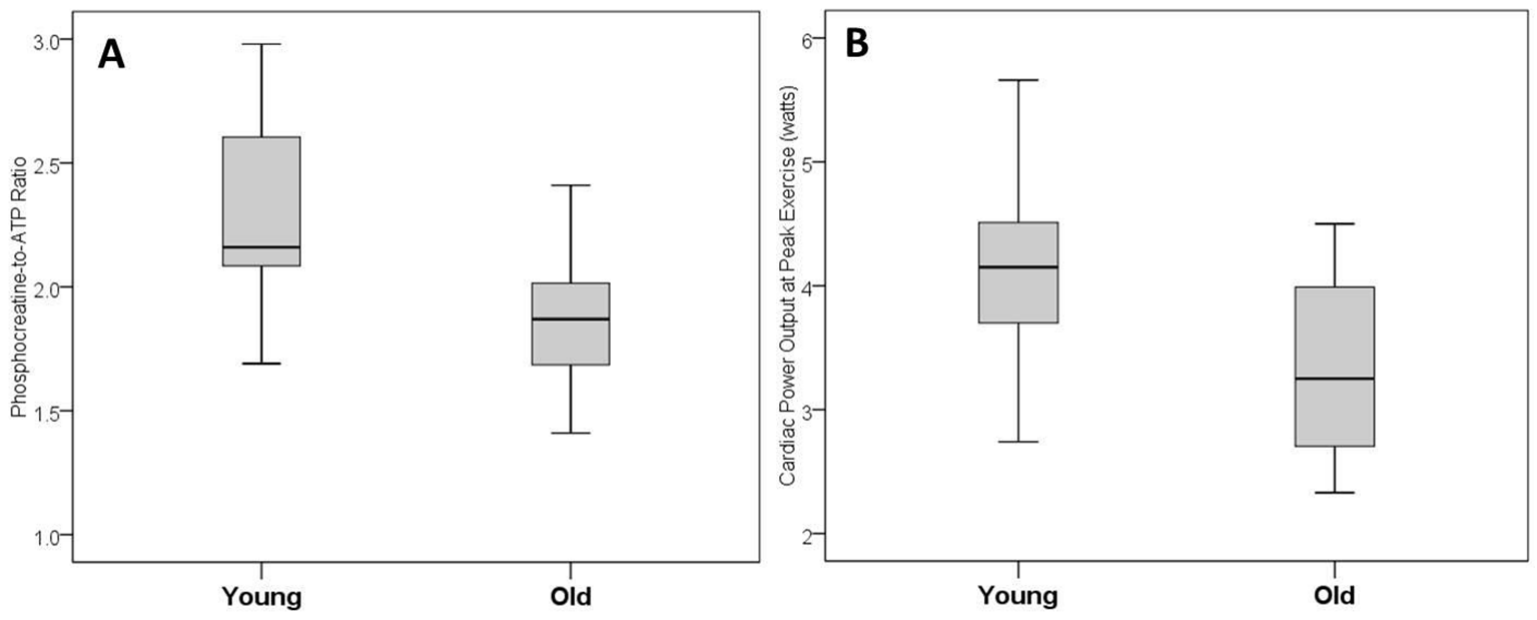
Cardiac high-energy phosphate metabolism—phosphocreatine-to-ATP ratio (A) and cardiac pumping capability—cardiac power output (B) in young and older women.

Cardiac MRI revealed that left-ventricular mass and early-to-late diastolic filling ratio were significantly lower in older women compared with younger women (78.6±9.7 vs 90.7±16.1 g p=0.01; and 1.3±0.5 vs 3.1±1.8, p<0.01, table 2). More importantly, older women demonstrated significantly lower PCr:ATP ratio compared with younger women (1.9±0.5 vs 2.3±0.6, p=0.03, table 2, figure 1A).

**Table 2:** Measures of cardiac structure, function and metabolism

	All participants	Young (≤50 years old, n=20)	Old (≥60 years old, n=15)	p Value
Left ventricular mass (g)	85.5±14.9	90.7±16.1	78.6±9.7	0.01
Eccentricity ratio (g/mL)	0.8±0.1	0.8±0.1	0.8±0.1	0.16
Left-ventricular ejection fraction (%)	61.7±7.7	58.7±7.6	65.8±5.8	0.01
PCr:ATP ratio	2.1±0.5	2.3±0.6	1.9±0.5	0.03
Longitudinal shortening (%)	19.4±5.9	19.4±3.0	19.5±4.9	0.92
E:A ratio	2.3±1.7	3.1±1.8	1.3±0.5	0.00

E;A, early-to-late diastolic  filling ratio; PCr:ATP, phosphocreatine-to-ATP.

Resting measures taken prior to cardiopulmonary exercise test revealed that metabolic measures that is, oxygen consumption and respiratory exchange ratio, were not significantly different between young and old groups (p>0.05, table 3). Resting cardiac variables such as stroke volume and cardiac output were significantly lower in the older age group (70.3±7.9 vs 87.1±14.4 mL/beats, p<0.01 and 4.9±0.8 vs 61.1±1.2 L/min, p<0.01). Older women also demonstrated higher systolic blood pressure and systemic vascular resistance (142.9±12.5 vs 128.5±16.8 mm Hg, p=0.01 and 1765±336 vs 1352±266 dyne/s/cm^5^, p<0.01; table 3).

**Table 3:** Resting gas exchange and central haemodynamics measures

	All participants	Young (≤50 years old, n=20)	Old (≥60 years old, n=15)	p Value
Resting metabolic variables				
Oxygen consumption (mL/min)	244.3±35.9	250.2±40.9	236.5±27.4	0.20
Oxygen consumption (mL/kg/min)	3.7±0.7	3.7±0.9	3.7±0.5	0.81
Respiratory exchange ratio	0.9±0.1	0.9±0.1	0.9±0.1	0.90
Resting haemodynamics				
Heart rate (beats/min)	69.9±7.6	69.6±7.6	70.3±7.9	0.80
Stroke volume (mL/beat)	80.1±16.2	87.1±14.4	70.8±14.1	0.00
Stroke volume index (mL/beat/m^2^)	46.2±8.3	49.4±8.0	42.1±6.8	0.01
Cardiac output (L/min)	5.6±1.2	6.1±1.2	4.9±0.8	0.00
Cardiac index (L/min/m^2^)	3.2±0.6	3.4±0.6	2.9±0.4	0.00
Systolic blood pressure (mm Hg)	134.7±16.6	128.5±16.8	142.9±12.5	0.01
Diastolic blood pressure (mm Hg)	86.0±9.9	85.6±9.9	86.7±10.1	0.75
Mean arterial blood pressure (mm Hg)	102.2±10.7	99.9±10.7	105.4±10.2	0.13
Cardiac power output (W)	1.3±0.3	1.4±0.3	1.2±0.2	0.06
Cardiac power output index (W/m^2^)	0.7±0.2	0.8±0.2	0.7±0.1	0.13
Systemic vascular resistance (dyne/(s·cm^5^))	1529.0±359.0	1352.1±265.6	1764.9±336	0.00

**Figure 2:**
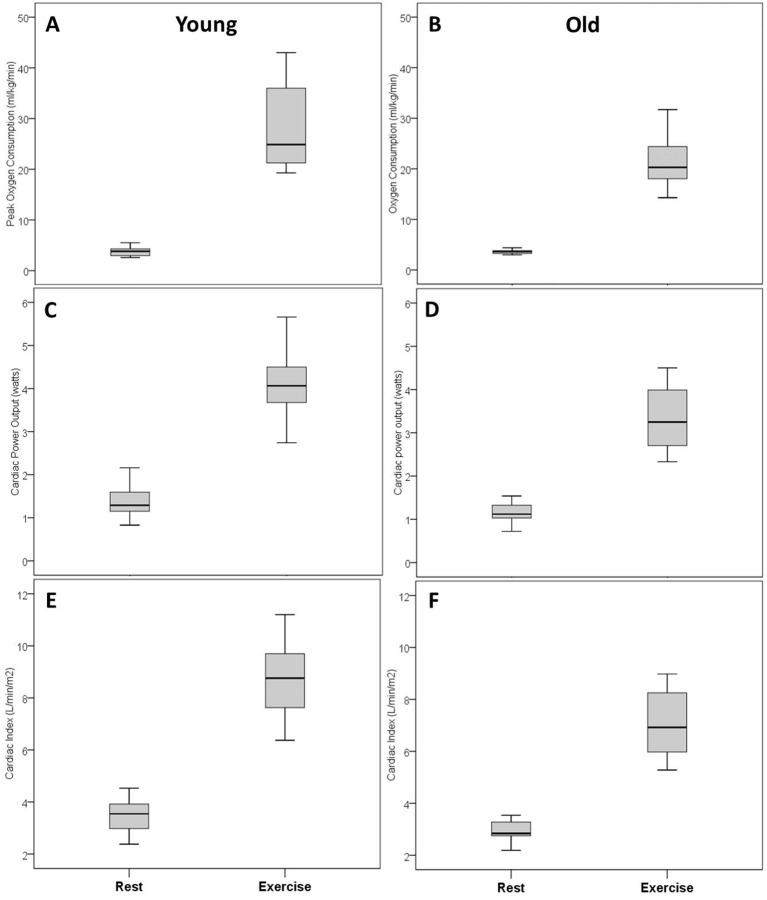
Cardiac and metabolic responses from rest to peak exercise in younger and older women: Oxygen consumption in young (A) and old (B); cardiac power output in young (C) and old (D); cardiac index in young (E) and Old (F).

**Figure 3:**
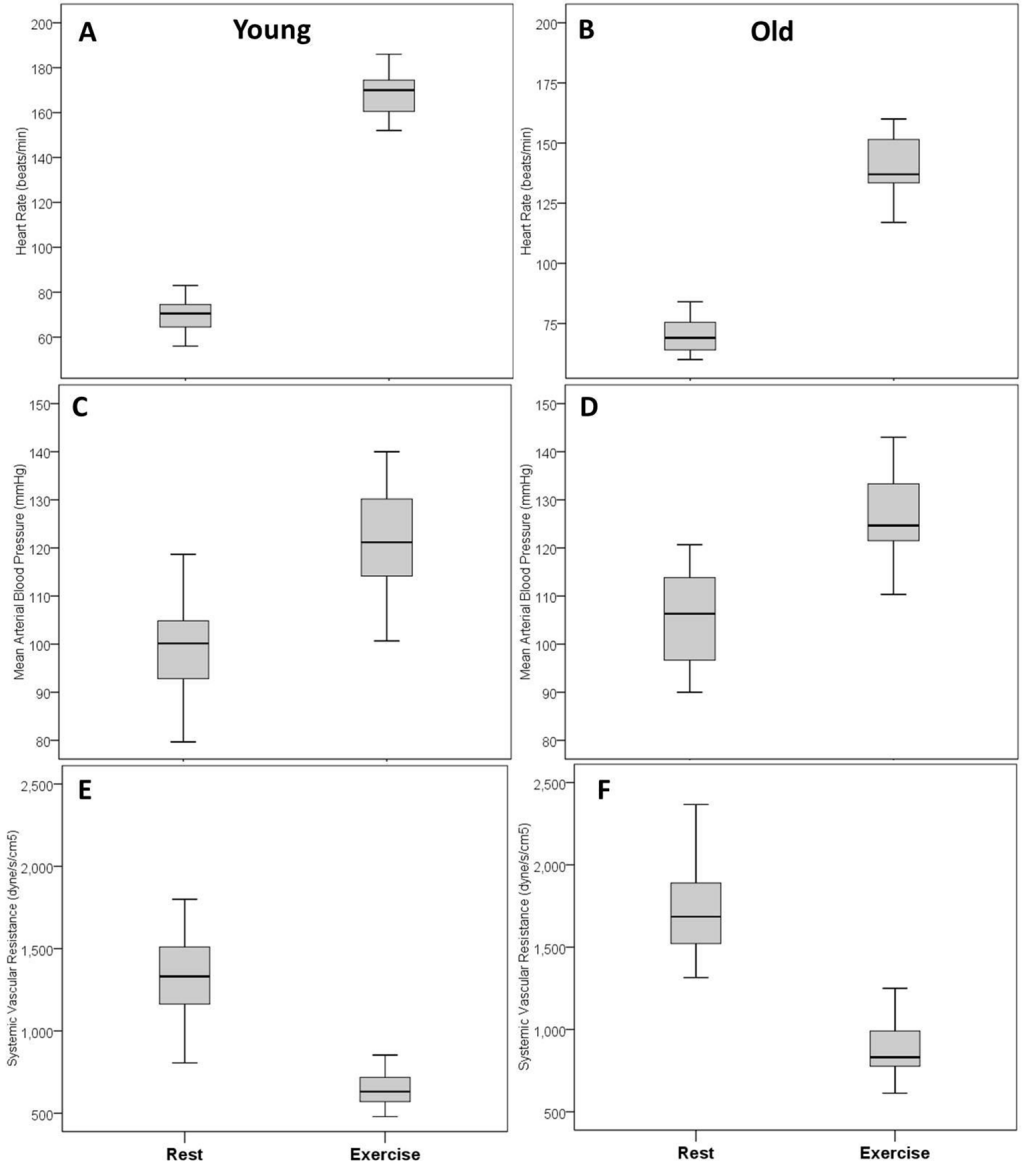
Cardiac responses from rest to peak exercise in younger and older women: Heart rate in young (A) and old (B); mean arterial blood pressure in young (C) and old (D); systematic vascular resistance in young (E) and old (F).

At peak exercise, older women exhibited significantly lower oxygen consumption compared with younger women (21.5±4.6 vs 28.6±8.8 mL/kg/min, p<0.01, table 4). Older women also showed significantly lower peak heart rate (140±15 vs 169±10 beats/min, p<0.01), peak cardiac output (11.9±2.3 vs 15.4±3.0 L/min, p<0.01) and peak cardiac power output (3.3±0.7 vs 4.1±0.8 W, p=0.01, table 4, figure 1B). Figures 2 and 3 present box-plots (median, first and third quartiles) for the rest and peak exercise variables, that is oxygen consumption, cardiac power output, cardiac index, heart rate, mean arterial blood pressure and systemic vascular resistance.

**Table 4:** Peak exercise gas exchange and central haemodynamic measures

	All participants	Young (≤50 years old, n=20)	Old (≥60 years old, n=15)	p Value
Peak exercise metabolic variables				
Oxygen consumption(mL/min)	1701.4±458.9	1940.3±434.4	1382.9±255.0	0.00
Oxygen consumption (mL/kg/min)	25.5±8.0	28.6±8.8	21.5±4.6	0.00
Arterial-venous oxygen difference (mLO_2_)	12.5±2.9	12.9±3.4	11.9±2.2	0.31
Respiratory exchange ratio	1.2±0.1	1.2±0.1	1.2±0.1	0.55
Peak exercise haemodynamics				
Heart rate (beats/min)	156.5±19.5	169.2±10.4	139.6±15.4	0.00
Stroke volume (mL/beat)	89.5±19.0	92.5±20.0	85.6±17.5	0.29
Stroke volume index (mL/beat/m^2^)	51.8±9.9	52.5±10.9	50.8±8.7	0.63
Cardiac output (L/min)	13.9±3.2	15.4±3.0	11.9±2.3	0.00
Cardiac index (L/min/m^2^)	8.0±1.8	8.8±1.7	7.1±1.3	0.00
Systolic blood pressure (mm Hg)	188.9±20.3	182.5±21.1	197.6±16.0	0.03
Diastolic blood pressure (mm Hg)	90.9±11.3	90.5±12.7	91.4±9.4	0.81
Mean arterial blood pressure (mm Hg)	123.6±10.1	121.1±10.2	126.8±9.3	0.10
Cardiac power output (W)	3.8±0.9	4.1±0.8	3.3±0.7	0.01
Cardiac power output index (W/m^2^)	2.2±0.5	2.4±0.5	2.0±0.4	0.03
Cardiac reserve (W)	2.5±0.9	2.8±0.9	2.2±0.8	0.05
Systemic vascular resistance (dyne/(s·cm^5^))	750.9±191.8	651.2±137.8	883.8±174.6	0.00

### Relationship between age and cardiac metabolism, function and performance

Age was significantly associated with decline in PCr:ATP ratio (r=−0.40, p=0.03), early-to-late diastolic filling ratio (r=−0.74, p<0.01) and cardiac output (r=−0.35, p=0.04). There was a significant positive relationship between age and resting systolic blood pressure (r=0.47, p=0.02) and systemic vascular resistance (r=0.42, p=0.03).

At peak exercise, age was significantly associated with lower heart rate (r=−0.82, p<0.01), cardiac output (r=−0.60, p<0.01), cardiac power output (r=−0.44, p<0.01) and oxygen consumption (r=−0.70, p<0.01). There was however a positive relationship between age and peak exercise systolic blood pressure (r=0.38, p=0.04) and systemic vascular resistance (r=0.64, p<0.01).

### Relationship between cardiac metabolism and cardiac function and performance

**Figure 4:**
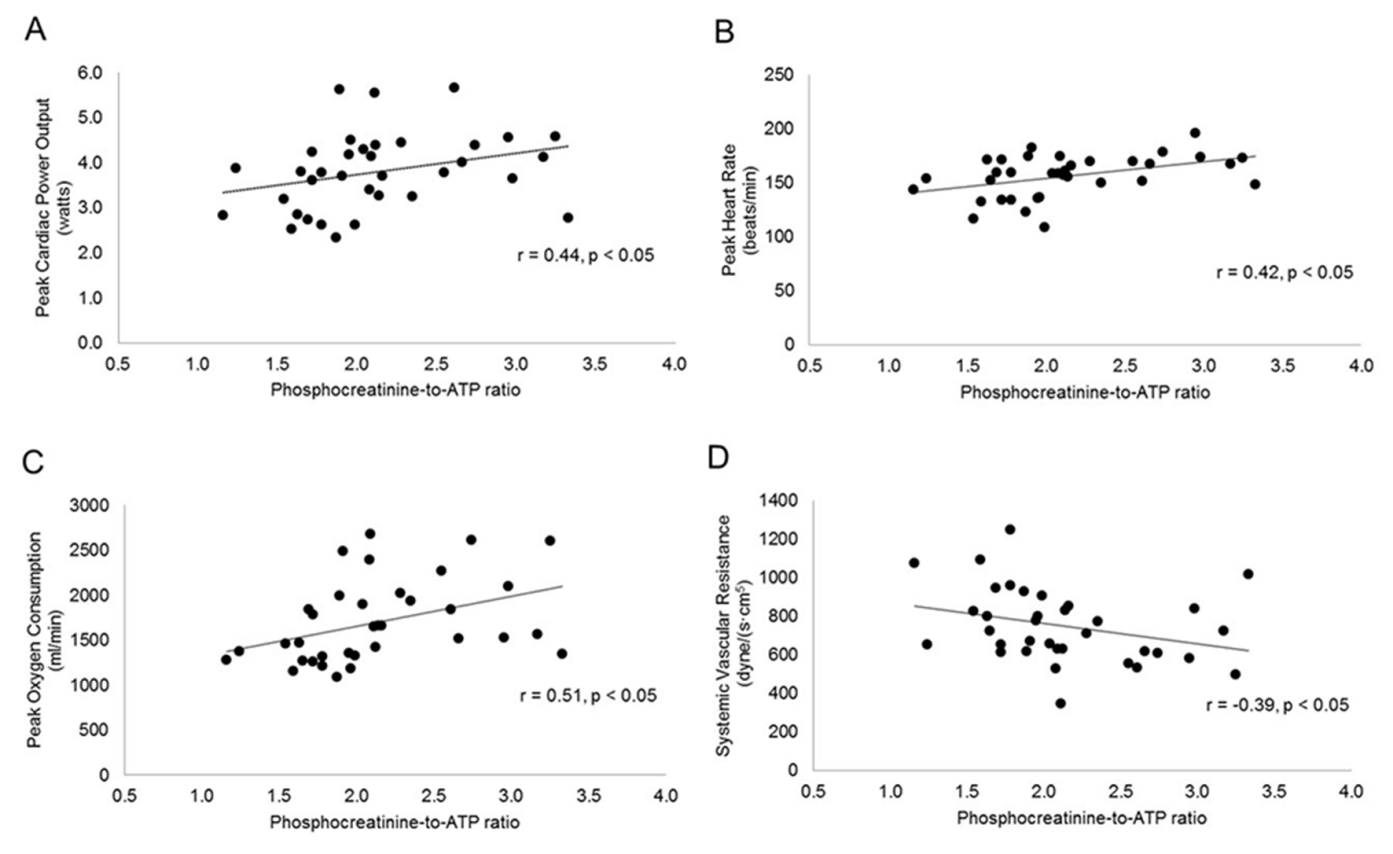
Relationship between cardiac high-energy phosphate metabolism and measures of cardiac function and performance obtained at peak exercise: Cardiac power output (A); heart rate (B); oxygen consumption (C) and systemic vascular resistance (D).

There was no significant relationship between PCr:ATP ratio and measures of cardiac structure and function, except for early-to-late diastolic filling ratio as a measure of diastolic function (r=0.46, p=0.02). Further analysis revealed a significant relationship between PCr:ATP ratio and measures of cardiac performance, that is peak cardiac power output (r=0.44, p=0.02, figure 4A), peak heart rate (r=0.42, p=03, figure 4B), peak oxygen consumption (r=0.51, p=0.01, figure 4C), cardiac output (r=0.45, p=0.01) and cardiac reserve (r=0.42, p=0.03). There was a significant negative relationship between PCr:ATP ratio and systemic vascular resistance (r=−0.39, p=0.04, figure 4D). PCr:ATP ratio was significantly correlated with systolic blood pressure at rest (r=−0.36, p=0.04), but not at peak exercise (r=−0.22, p=0.09).

## Discussion

This is the first study to address the relationship between high-energy phosphate metabolism and maximal performance of the heart assessed in response to cardiopulmonary exercise testing. The study is clinically relevant as it helps better understand the role of high-energy phosphate metabolism in overall function and performance of the heart, further supporting previous suggestion that diminished cardiac metabolism is a potential mechanisms responsible for the development of cardiac dysfunction and heart failure.^1^ The major finding of this study suggests that cardiac high-energy phosphate metabolism, as measured by PCr:ATP ratio, is significantly correlated with measures of diastolic function and performance of the heart. In addition, the study findings demonstrate that cardiac metabolism, function and performance of the heart decline with age.

Results from previous studies evaluating changes in cardiac metabolism with age are conflicting. Okada *et al* and Köstler *et al* 18 19 reported significant decline in PCr and ATP levels in older subjects compared with younger subject. However, a decline in the PCr:ATP ratio was not observed. Our findings support recent studies reporting that PCr:ATP ratio decreases with age,^11 17 20^ that is older women demonstrate significantly lower PCr:ATP ratio compared with younger women and that this decline in cardiac metabolism is negatively correlated with age. The decline in cardiac high-energy phosphate metabolism possibly reflects a number of age-associated changes including a decrease in mitochondrial oxidative phosphorylation, impaired myocardial lysosomes and decline in creatine kinase activity with age.^21^ Oxidative phosphorylation is responsible for 95% of cardiac ATP production; thus, a decline in oxidative phosphorylation will lead to the decrease in overall cardiac high-energy metabolism. Creatine kinase is a catalytic enzyme that is crucial in the regeneration of ATP from ADP and PCr, and its decline with age may lead to subsequent decrease in PCr level.^18^ Previous studies also reported an age-associated elevated increase in lipofuscine and decreased lysosomal efficiency leading to subsequent death of cardiac myocytes.^17^ Kates *et al* 22 also reported a decline in myocardial fatty acid utilisation and oxidation with no change in glucose utilisation, which consequently lead to diminished overall cardiac high-energy phosphate metabolism.

Measures of cardiac function and performance change with age that is, decreased early-to-late diastolic filling ratio, cardiac output and cardiac power output, whereas systemic blood pressure and left-ventricular ejection fraction increased. These age-related haemodynamic changes have been previously reported. The increase in systolic blood pressure is due to accumulation of collagen and calcium in the large arteries, along with loss of elastic fibres from the medial layer.^22 23^ The thickening and stiffening of large arteries increase systemic vascular resistance, thus leading to raised systolic blood pressure. A decrease in early-to-late diastolic filling ratio with age occurs due to age-associated decline in left-ventricular early diastolic filling rate and increase in late diastolic ventricular filling.^24^ Early diastolic filling rate has been observed to decline progressively after the age of 20 and diminished to 50% by 80 years of age.^24^ An increase in late diastolic ventricular filling with age occurs due to increased atrial contribution to ventricular filling.^25^ Also, decline in diastolic function occurs due to a number of age-associated changes such as myocardial collagen accumulation, matrix proliferation, reduced calcium overload threshold and reduced ventricular length.^26^ These changes increase wall stress and reduce cardiac contractility, leading to subsequent decline in diastolic function.

This study shows that cardiac high-energy phosphate metabolism has a positive and significant relationship with early-to-late diastolic filling ratio, peak cardiac output, peak cardiac power output and cardiac reserve. This suggests that a decline in cardiac high-energy phosphate metabolism might lead to age-related decline in cardiac function and performance. It should also be noted that PCr:ATP ratio was negatively correlated with systolic blood pressure and systematic vascular resistance. As blood pressure is an important determinant of cardiac energy consumption, it potentially may explain observed reduction in PCr:ATP ratio in older women, as previously suggested.^27^

This is the first human study to define the relationship between high-energy metabolism and overall maximal function and pumping capability of the heart represented by peak cardiac power. Peak cardiac power has been proposed to be the best indicator of overall cardiac function because it integrates both flow-generating and pressure-generating capacities of the heart, and as a such has been shown to be the strongest predictor of prognosis in heart failure.^11^

Our findings therefore support previous theory suggesting that diminished cardiac metabolism may play a significant role in mechanisms of development of cardiac dysfunction and heart failure.^1^ Previous animal studies have shown that alterations in cardiac high-energy phosphate metabolism lead to reduced contraction velocity, diminished systolic function and reduced cardiac contractility.^6 7^ However, the exact mechanism of how cardiac metabolism leads to diminished cardiac function and performance still need to be defined. Fundamentally, ATP hydrolysis is a key in driving the myosin head sliding movement along actin filament during contraction.^28^ Diminished cardiac high-energy phosphate metabolism will lead to reduced cardiac high-energy phosphates. The rates of myocardial ATP production must closely matched the cardiac metabolic demand. A halt in ATP production would deplete cardiac ATP stores in mere 15 s.^2^ Therefore, decline in ATP production may affect cardiac contractility and viability and highly likely cause diminished cardiac function and performance. Moreover, a decline in PCr level might disrupt cardiac contractility. During inadequate oxygenation, PCr and ADP is rapidly converted into ATP through the rapid and reversible creatine kinase reaction.^29^ This is essential to maintain high levels of ATP and low levels of ADP. When energy demand outweighs supply, PCr level falls to maintain normal levels of ATP, with an increase in ADP level.^5 29^ Elevated free ADP inhibits intracellular enzymes and disrupts cardiac contractile function.

This study is not without limitations. Only women were studied due to the significant differences in age-associated changes in cardiac morphology and function between men and women as previously confirmed.^9^ Further larger mechanistic studies to include both women and men are required to provide better understanding of the interaction between cardiac energetics and function.

## Conclusion

High-energy phosphate metabolism and performance of the heart decline with age. A significant positive relationship between PCr:ATP ratio and peak cardiac power output suggests that cardiac high-energy phosphate metabolism may be an important determinant of cardiac function and performance.

**Key messages**

**What is already known on this subject?**

Age-related decline in high-energy phosphate metabolism and cardiac performance may play an important role in the development of cardiac dysfunction and heart failure.

**What might this study add?**

This study demonstrates a significant positive relationship between phosphocreatine-to-ATP ratio and peak cardiac power, suggesting that cardiac high-energy phosphate metabolism may be an important determinant of cardiac function and performance.

**How might this impact on clinical practice?**

Therapeutic interventions known to improve cardiac metabolism may lead to improvement in overall cardiac function.
